# Detection of *Trypanosoma evansi* in a naturally infected cat in Indonesia using bioassay and molecular techniques

**DOI:** 10.14202/vetworld.2023.828-833

**Published:** 2023-04-20

**Authors:** Dwi Priyowidodo, Ana Sahara, Joko Prastowo, Wisnu Nurcahyo, Lintang Winantya Firdausy

**Affiliations:** Department of Parasitology, Faculty of Veterinary Medicine, Universitas Gadjah Mada, Yogyakarta, 55281, Indonesia

**Keywords:** bioassay, feline, internal transcribed spacer-1 molecular detection, *Trypanosoma evansi*

## Abstract

**Background and Aim:**

The prevalence of surra in domestic cat is seldom and it is caused by *Trypanosoma brucei* and *Trypanosoma evansi*. However, molecular diagnostic approaches are required owing to similarities in their morphology. In Yogyakarta, a domestic cat was diagnosed with trypanosomiasis; however, the causative species was undetermined. Therefore, we aimed to molecularly and biologically identify the isolate.

**Materials and Methods:**

Approximately 1 mL of blood from an infected cat was collected into EDTA tube and separated for inoculation into donor mice, blood smear, and DNA isolation. Two donor mice was then used for increasing the number of parasite in order to infect 10 experimental mice. Parasitemia was monitored daily in each experimental mouse by preparing a wet mount and Giemsa-stained thin blood smear. The blood of experimental mice that reached the peak of parasitemia was then collected and used for DNA isolation. Each blood sample, which collected from infected cat and experimental mice, was then isolated and amplified the DNA by polymerase chain reaction using ITS-1. The parasitemia pattern and viability of the animals were observed to determine the biological characteristics of trypanosomatid, while to assess the molecular characteristics, the internal transcribed spacer (ITS)-1 amplification was used.

**Results:**

The prepatent period of this trypanosomatid is between 2 and 4 dpi, whereas the life span of mice is approximately 4–10 dpi. Morphologically, the trypomastigote in the cat blood smear had long slender and intermediate shapes. However, only the long slender form was detected. Among the total of 410 nucleotides (NT) of ITS-1 sequences, 25 NT substitutions differed between the cat and mouse isolates. Phylogenetic analysis revealed that both samples had a close genetic relationship with *T. evansi*.

**Conclusion:**

*Trypanosoma evansi*, a highly virulent trypanosomatid, was isolated from a cat in Yogyakarta.

## Introduction

The occurrence of trypanosomiasis with natural infection in cats has seldom been recorded in the literature because cats are relatively more resistant than other vertebrates; hence, trypanosomiasis is infrequently [1]. Feline trypanosomiasis in domestic cats has been reported in one case in Canada and India [1, 2], three cases in Kuwait [3], and seven cases in Iraq [4]. The infection was caused by *Trypanosoma brucei* and *Trypanosoma evansi* [1–4], both of which elicit the same clinical symptoms in cats. Corneal opacity, edema around the eyes and head that continues into the abdomen, and lacrimation are the most common manifestations. The appearance of these clinical symptoms is directly proportional to parasitemia. The symptoms of parasitemia appeared moderate at the beginning of the first peak, such as loss of appetite, lethargy, coarse, dry hair, and fever. During the second peak of parasitemia, fever is followed by edema in one part of the eyelid, where the same changes always occur in every peak infection. Edema in the eyelid is always followed by corneal opacity, which reverts to normal with a decrease in the number of trypomastigotes in the blood smear. Both eyes eventually become fully infected after 7–8 peaks of infection, the corneal opacity transitions into permanent blindness, and the cat becomes lethargic, less active, and has a reduced appetite. In severe conditions, there is progressive paroxysm and emaciation, which eventually lead to death [1, 5].

Typically, trypomastigotes of trypanosomes are spindle-shaped cells with nuclei in the mid-body and kinetoplasts in the posterior region. This parasite contains a single flagellum derived from the flagellar pocket in the posterior body, extends to the anterior region, and appears as free flagella at the anterior end body [6]. Trypomastigote morphology can be used to identify several species of trypanosomes through their pleomorphic or monomorphic size, location of the kinetoplast, and shape of the posterior end. However, some species cannot be distinguished based on the trypomastigote morphology; these include *T. brucei* and *T. evansi* [7]. *Trypanosoma brucei* has pleomorphic trypomastigotes, starting from a short stumpy form, intermediate, and long slender form, whereas the trypomastigote of *T. evansi* only has a long slender form [8]. However, *T. evansi* trypomastigotes can reportedly be pleomorphic and affected by the growth phase of the parasite, the host immune response, and the infected host species [5, 8, 9]. Pleomorphic trypomastigotes of *T. evansi* have been reported in cats, monkeys, and one-humped camel blood film [5, 9]. Therefore, a molecular diagnostic approach is required to distinguish the causative organism. In addition to the molecular detection of *Trypanosoma* infection, polymerase chain reaction (PCR) and DNA sequence analyses have been widely used to establish a phylogenetic connection and to reveal trypanosomatid inter- and intra-specific genetic variants [4, 10–12]. The biological and genetic diversity and characteristics of *Trypanosoma* isolated from various vertebrates have been thoroughly reported and documented worldwide; however, information on the biological and genetic characteristics of feline trypanosomiasis remains limited.

The internal transcribed spacer (ITS) region is a valuable target for phylogenetic analysis, evaluation of evolutionary processes, and determination of taxonomic identities of protozoa, including *Trypanosoma* [13], particularly when the characteristic of parasite morphology are limited. These sequences could also be used to identify intra- and inter-specific variability in *Trypanosoma* spp., including *T. brucei brucei*, *Trypanosoma brucei gambiense*, *Trypanosoma rangeli*, and *T. evansi* from various vertebrates in several countries [13–15]. Therefore, this study aimed to identify and characterize the trypanosomes which derived from a naturally infected domestic cat in Yogyakarta, through bioassay and molecular approachment. The molecular analysis was based on PCR amplification and ITS-1 sequence analysis would help provide information about the trypanosome and estimate its phylogenetic position among other *Trypanosoma* spp.

## Materials and Methods

### Ethical approval

This study was approved by the Ethical Clearance Committee team of Faculty of Veterinary Medicine, Universitas Gadjah Mada (Number 0018/EC-FKH/Int/2020).

### Study period and location

This study was conducted from May to October 2020 at Parasitology Laboratory, Faculty of Veterinary Medicine, Universitas Gadjah Mada.

### Trypanosome sample and experimental animals

Blood was obtained from an infected cat diagnosed with trypanosomiasis based on the presence of trypomastigotes on the blood smear. According to Kurnia *et al*. [16], the cat showed mild blepharospasm with hyperlacrimation, conjunctivitis, aqueous flare, and turbidity in the anterior chamber, which led to the accumulation of fibrin that was seen floating in the aqueous humor which was bilaterally progressive. Other clinical changes that appear are decreased appetite, fever, pale mucosa, dehydration, and the presence of edema in the submandibular area to the shoulder. Approximately 1 mL of blood was collected from the cephalic vein and put into an ethylenediaminetetraacetic acid (EDTA) tube. The collected blood was then separated for inoculation into donor mice, blood smear, and DNA isolation.

To boost the number of parasites, two donor mice [Deutch Democratic Yokohama (DDY) strain] were used. The infected blood was diluted in physiological saline solution before being administered intraperitoneally in each mouse. Parasitemia was monitored daily by preparing wet mounts (data not shown). Each mouse with peak parasitemia (>15 *Trypanosoma* identified in one field of view at 10× magnification), was euthanized by placing them in a CO_2_ chamber. The infected blood was collected through the cardiac puncture into tubes containing 10% EDTA. The number of parasites in the blood of donor mice was counted using a Neubauer chamber and then diluted into 1 × 10^4^ trypanosomes/mL, with a pH of 8.0 using phosphate-buffered-saline-glucose (PBSG). The dilution results were then utilized to infect experimental mice.

Ten male DDY strain mice weighing between 25 and 30 g obtained from the Animal House Research Center of Universitas Gadjah Mada, Indonesia, were used in this study. The mice were housed in plastic cages under standard hygienic conditions, with wood shavings as bedding that was replaced weekly. The mice were fed twice daily with rat pellets and water ad libitum. The mice were acclimatized to these conditions for 1 week in the Parasitology Laboratory, Faculty Veterinary Medicine, Universitas Gadjah Mada, where the experiments were conducted. Preliminary data on body weight were collected twice during the acclimation process before inoculation with trypanosomes to ensure even distribution in the body weight of mice (data not shown).

In each experimental mouse, 0.2 mL of 10^4^ trypanosomes/mL dilution was injected intraperitoneally. Parasitemia was monitored daily by preparing a wet mount and Giemsa-stained thin blood smear. Daily parasitemia progression was categorized as described by Raina *et al*. [17]. Mice that reached the peak of parasitemia from each group were randomly selected to euthanize by placing them in a CO_2_ chamber and collecting their blood. The blood sample was then stored in a refrigerator for further DNA isolation.

### DNA extraction and amplification

A Thermo Scientific GeneJET Genomic DNA Purification Kit (Thermo Scientific^®^, Vilnus, Lithuania) was used for total DNA isolation from blood samples according to the manufacturer’s instructions. The DNA was stored at –20°C until further examination.

Amplification of target gene DNA was performed by PCR using ITS-1 (F) (5’-ACCT GCAG CTGG ATCA TTTT-3’) and ITS-1 (R) (5’- GCTG CGTT CTTC AACG AAAT-3’) primers from Budiati [18]. PCR was performed in 25 μL volume, including a 5 μL DNA template; a 12.5 μL of master mix (Go Taq^®^ Green Master Mix, Promega, Wisconsin, USA); 1 μL of each primer at a 10 pmol; and 5.5 μL ddH_2_O. The amplification reactions were as follows: 94°C for 4 min followed by 35 cycles of 94°C for 30 s, 55°C for 30 s, 72°C for 30 s, and a final extension was 72^o^C for 5 min.

### Sequencing and phylogenetic analysis

The purified PCR product was sequenced using first Base Sequencing (Malaysia), and analysis was performed using the MEGA X program (NCBI, USA). Subsequently, the sequences were edited, followed by the multiple alignments of the sequence data in NCBI GenBank, USA. All nucleotides (NT) were analyzed to determine genetic relationship using the Kimura 2-parameter method with 1000 bootstrap replicates. In addition, phylogenetic analysis was performed using maximum likelihood to depict the relationship between the species.

### Statistical analysis

Data were analyzed using descriptive statistics.

## Results

### Biological characteristics

**Figure-1:**
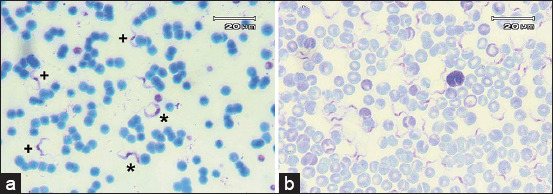
Morphology of trypomastigote in the blood film. (a) Blood film of infected cat; (b) Blood film of experimental mice infected with an isolate from the cat; (*) intermediate form; (+) long slender form.

Blood smears from each host demonstrated different trypomastigote morphologies. There were two shapes, long slender and intermediate forms, of trypomastigotes in the feline blood film. Notably, the average width of the intermediate form (2.8 ± 0.1 μm) was approximate twice the width of the slender form (1.6 ± 0.2 μm) despite similar average lengths. In contrast, the trypomastigotes appear uniformly long and slender in experience mice blood film. The differences in the shape and size of trypomastigotes in each blood film sample are presented in Figure-1 and Table-1, respectively.

**Table-1:** Size of trypomastigote in the blood film of each sample.

Sample	The size of trypomastigote (µm)		Average (µm)

	Length	Width	
A	26.5	1.4	L: 25.2 ± 2.5 W: 1.6 ± 0.2
	26.8	1.5	
	22.3	1.8	
A*	29.1	2.8	L: 29.6 ± 1.0 W: 2.8 ± 0.1
	28.9	2.7	
	30.8	2.9	
B	17.3	1.8	L: 17 ± 0.5 W: 1.8 ± 0.1
	16.5	1.7	
	17.3	1.8	

A=Trypomastigote (slender form) in blood film of infected cat, A*=Trypomastigote (intermediate form) in blood film of infected cat, B=Trypomastigote in blood film of experimental mice

At 1–4 dpi (average, 2.8 days), the first trypomastigote was observed in wet mounts of experimental mouse blood or the prepatent period. The peak of parasitemia occurs between 1–4 days after the first trypomastigote was identified in the wet mount blood or within 2–8 dpi. The mice eventually died without presenting any clinical symptoms. Hence, parasitemia has just one wave. The progression of parasitemia in each experimental mouse is shown in Table-2.

**Table-2:** Daily parasitemia progression in experimental mice.

No. of mice	Dpi 1	Dpi 2	Dpi 3	Dpi 4	Dpi 5	Dpi 6	Dpi 7	Dpi 8
1	0	0	+	++	++	+++	++++	dead
2	0	+	+	+	+++	++++	dead	
3	0	0	+	++++	++++	dead		
4	+	++	+++	+++	++++	dead		
5	0	0	+	+++	++++	dead		
6	0	0	+	++	+++	++++	dead	
7	0	+	+	+++	++++	dead		
8	+	+	++	++++	dead			
9	0	+	++	+++	++++	dead		
10	0	0	+	+	++	++++	dead	

Dpi=Days post-infection

### Molecular diversity

**Figure-2:**
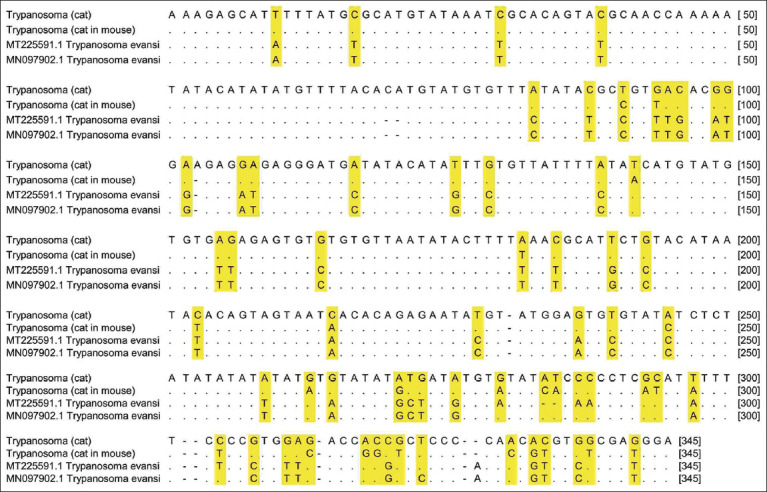
Comparison of the sequences in the ITS-1 region between the samples and the references from GenBank. The differences between each nucleotide are highlighted.

Among the total of 410 NTs of ITS-1 sequences, 25 NT substitutions differed between the samples. The locations of the NT variation sequences are displayed in Figure-2. The BLAST data of cat sample exhibited 92.92% homology with *T. evansi* dog isolate from India (MN097902), whereas the homology of the mouse sample was 95.04% with *T. evansi* buffalo isolates from India (MT225591).

**Figure-3:**
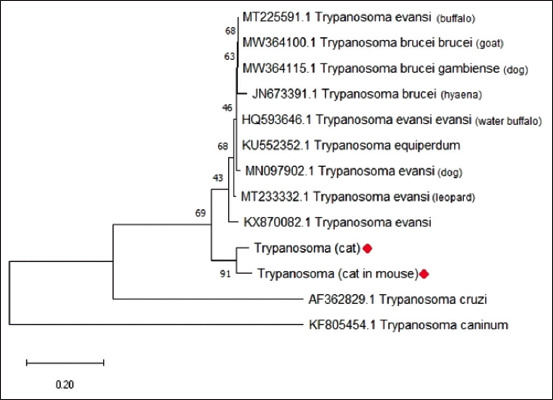
Phylogram analysis of internal transcribed spacer-1 sequences, with maximum likelihood algorithm using the Kimura parameter, depicting the relationship between the isolates and the references sequences in GenBank. ♦Represent isolates in the present study.

The phylogenetic analysis of the samples based on the ITS-1 sequence is depicted in Figure-3. Based on the ITS-1 sequence, both samples were in the same monophyletic group or clade as *T. evansi* (KX870082).

## Discussion

Indonesia is a surra-endemic area. In most cases of surra incidence in Indonesia, the infection is transmitted to the livestock by biting flies such as *Tabanus* spp., *Haematopota* spp., *Chrysop* spp., *Stomoxys* spp., and *Haematobia* spp. [19]; however, carnivores could also be infected by eating carcasses of infected animals. Therefore, we initially suspected that the infected cat in our study might have been infected with *T. evansi*. However, we could not obtain more information about its history because it was a feral cat. Therefore, we undertook a diagnostic approach using bioassays and molecular techniques. Trypomastigotes were detected in two morphologies in the cat blood film: Intermediate and slender, but only a long slender blood smear was observed in both experimental mice.

Misra *et al*. [5] reported that host differences affect *T. evansi* trypomastigote. They will have the pleomorphic form (slender, intermediate, and stumpy) when infect a domestic cat, whereas rodent hosts adopt a monomorphic (slender only) form. Pleomorphism is the manifestation of metabolic adaptability when the parasite is against the host immune response in acute infection, and it fades in chronic infection due to the incapacity of the host to battle the parasitic burden [5, 20].

In contrast to the incidence in cats, trypanosomiasis in mice does not cause any clinical symptoms. However, death always follows the infection process, depending on the mouse strain and the virulence of the parasite [21, 22]. The virulence of parasites is their capacity to cause diseases and damage the host. Subekti *et al*. [22] and Kamidi *et al*. [23] reported that *Trypanosoma* virulence could be observed based on its biological characteristics, such as the pattern of parasitemia and host survival ability, against infection. *Trypanosoma* virulence in Indonesia can be classified into three biotypes. Biotype 1 (high virulence) has a parasitemia pattern that increases rapidly. Therefore, the ability of the host to survive infection is likewise limited (mice die in less than 7 dpi). Biotype 2 (moderate virulence) is characterized by the presence of undulant parasitemia, and mice die 7–15 dpi. In contrast, biotype 3 (low virulence) is characterized by persistent parasitemia (mice will die in more than 15 dpi) [22, 24].

In regard to our findings, trypanosomes were classified as biotype 1 and had high virulence because they were due to a single wave of parasitemia and short host survival. *Trypanosoma* with high virulence will cause the energetic cost of mounting an immune response for an already damaged animal to be too high, resulting in a hyperacute infection process in which parasitemia will increase rapidly, followed by death after 10–12 days post-infection, without causing any clinical changes [21].

Njiru *et al*. [25] reported that the ITS-1 region could be used to distinguish trypanosome species based on product lengths produced by each species of trypanosomes. However, *T. evansi* and *T. brucei* ITS-1 fragments have the same base pair size [26], and most of the published data also indicate that the efficacy of ITS-1 is low for trypanosomes discrimination and less sensitive [13, 14, 27].

In our study, each sample was similar to several *T. evansi* isolates from India and Africa, followed by their genetic diversity. This is in accordance with the findings of Sarkhel *et al*. [28], who stated that the ITS-1 region is suitable for analyzing the genetic diversity of *T. evansi* in different regions and hosts. Hamilton *et al*. [29] reported that the adjustment mechanism for the host (host fitting) is the primary evolutionary mechanism in *Trypanosoma*. *Trypanosoma evansi* can adapt to different hosts and ecologies, giving rise to genetic diversity [30]. Sarkhel *et al*. [28] reported that the genetic diversity of *T. evansi* was caused by the co-evolution of the parasite with the host and vector. Tian *et al*. [31] and Areekit *et al*. [14] demonstrated that the robustness of phylogenetic trees using the ITS-1 region is low. This may be attribute to the limited length of the ITS-1 NT sequence; hence, the entire ITS region was preferable. In contrast to the previous report, the bootstrap value in the phylogenetic analysis of this study was relatively high (>50). This may also be attributed to the limited sequence data of feline trypanosomiasis in the gene bank. However, the molecular characteristics of the cat isolate require further studies using other sequence data.

## Conclusion

We identified *T. evansi* infection, which was obtained from a cat in Yogyakarta; the trypanosome has high virulence and is included in biotype 1. The genetic diversity between the samples, as well as in the GenBank data, might be influenced by the host factors and the limited length of ITS-1 NT sequence. Due to its rarity, molecular characteristics must be further elucidated to facilitate the detection of the parasite at the species scale.

## Authors’ Contributions

DP: Managed and supervised the study. AS, JP, WN, LWF: Collected, recorded, and analyzed the samples. DP and LWF: Interpreted the data and drafted and revised the manuscript. All authors have read, reviewed, and approved the final manuscript.
